# Quality of life, physical symptoms, and psychological symptoms according to the status of chronic vestibulopathy

**DOI:** 10.1371/journal.pone.0312727

**Published:** 2024-11-04

**Authors:** Sang-Yoon Han, Sang-Yeon Lee, Myung-Whan Suh, Jun Ho Lee, Moo Kyun Park

**Affiliations:** 1 Department of Otolaryngology-Head and Neck Surgery, College of Medicine, Hanyang University, Seoul, Republic of Korea; 2 Department of Otorhinolaryngology-Head and Neck Surgery, Seoul National University Hospital, Seoul, Republic of Korea; 3 Sensory Organ Research Institute, Seoul National University, Medical Research Center, Seoul, Republic of Korea; UFPE: Universidade Federal de Pernambuco, BRAZIL

## Abstract

**Objectives:**

Symptomatic vestibulopathy impairs patients’ lives. However, few studies have explored the lives of patients with compensated or asymptomatic vestibulopathy. This study investigated the quality of life (QOL), psychological health, and physical function of patients with vestibulopathy.

**Materials and methods:**

Using the eighth Korea National Health and Nutrition Examination Survey database, we included individuals with data on demographic factors, diabetes, hypertension, dizziness experiences, pure-tone audiometry, video head impulse test (vHIT), Health-related Quality of Life Instrument with 8 Items, General Anxiety Disorder 7-item scale, stress, and walking and sitting times. Participants were classified into the following groups: an uncompensated group with abnormal vHIT result and chronic dizziness, a compensated group with abnormal vHIT result and a history of dizziness, an asymptomatic group with abnormal vHIT result and no history of dizziness, and a normal group without abnormal vHIT result or a history of dizziness.

**Results:**

Uncompensated vestibulopathy was more common in older individuals and women. The uncompensated group showed impairments in climbing stairs (*P* < 0.001), pain (*P* < 0.001), vitality (*P* = 0.001), working (*P* < 0.001), depression (*P* < 0.001), sleep (*P* = 0.001), happiness (*P* = 0.002), anxiety (*P* = 0.006), and stress (*P* = 0.003). The compensated group showed deficits in pain (*P* < 0.001), work (*P* = 0.006), sleep (*P* = 0.001), and happiness (*P* = 0.001). The asymptomatic group had no deficits in QOL, psychological health, or physical function. These tendencies were similar after controlling for age and gender.

**Conclusion:**

Vestibulopathy with a history of dizziness has a long-lasting impact on QOL and emotional status, even after compensation. Uncompensated vestibulopathy has a significant effect on QOL and mental health. Notably, though, the compensated group also showed a reduction in QOL. Appropriate interventions for each category of patients should be provided based on their impaired functions.

## Introduction

Dizziness can be induced by many diseases, such as benign paroxysmal positional vertigo (BPPV), acute unilateral vestibulopathy (AUVP), Meniere’s disease, vestibular migraine, and orthostatic hypotension [1]. These diseases have distinct etiologies and can be diagnosed by synthesizing the results of vestibular function tests [1]. The diseases tend to provoke recurring symptoms, which can affect patients’ lives significantly [1, 2].

Some individuals with vertigo do not consume enough food due to nausea and vomiting, and their dizziness can make it difficult to work or engage in activities [1, 2]. In addition to increasing their fall risk [3], chronic dizziness may heighten a patient’s sense of depression and anxiety due to a fear of recurrent dizziness and associated symptoms such as nausea, vomiting, and headache [4]. Therefore, managing dizziness and preventing its recurrence are important for improving a patient’s quality of life (QOL).

Fortunately, most BPPV patients stabilize after successful canalith repositioning therapy [1]. However, AUVP and Meniere’s disease may provoke sequelae for the vestibular organs [1, 5, 6] and lead to lifelong issues with sustained imbalance and mild dizziness [5, 6]. To overcome the symptoms induced by uncompensated vestibulopathy, vestibular rehabilitation can be applied to help patients improve imbalance and mild chronic dizziness with somatosensory and visual support [7]. Although vestibular rehabilitation alone cannot guarantee normal-range vestibular test results, it can improve patients’ symptoms and QOL [7]. However, some patients report chronic dizziness despite undergoing vestibular rehabilitation, indicating uncompensated issues [6].

Several studies have assessed patients’ QOL and emotional status [4, 8]. These studies have shown that chronic dizziness has a negative effect on QOL, depressive moods, and anxiety. However, no study has yet compared normal individuals and those with chronic uncompensated vestibulopathy, compensated vestibulopathy, or chronic non-symptomatic vestibulopathy. The goal of the current study was to evaluate participants’ QOL, physical performance, and emotional status based on their vestibulopathy status, with a particular focus on compensated vestibulopathy.

## Materials and methods

### Subject inclusion and data extraction

The Korea National Health and Nutrition Examination Survey (KNHANES) was initiated in 1998 and has been conducted annually since 2007 to evaluate the health and nutritional status of the general population by the Korea Disease Control and Prevention Agency, which is an organization under the Korean government. The physical and laboratory examinations have been conducted in the mobile examination vehicle, and examinations for otolaryngologic diseases have also been included. The types of examinations vary each year. The vHIT was performed only in 2021. We utilized data gathered in 2021 from the eighth KNHANES, which was conducted after obtaining written informed consent from all participants and receiving approval from the Institutional Review Board of Korea Disease Control and Prevention Agency (IRB no. 2018-01-03-5C-A). The 8^th^ KNHANES survey was carried out based on the relevant guidelines and regulations, and our study was designed in accordance with the STROBE statement. This survey used two-stage stratified sampling methods to select samples from the general population in the Republic of Korea. Data from other years of the eighth KNHANES were excluded due to a lack of video head impulse test (vHIT) results. Among the individuals surveyed, we extracted data for those who had vHIT results. Afterward, to exclude dizziness attributable to causes other than vestibular issues, we screened out individuals with a history of dizziness but without a catch-up saccade (CUS) or an abnormal vestibulo-ocular reflex (VOR) in vHIT. For those finally selected as participants, we extracted data on demographic factors, household income, history of dizziness, pure tone audiometry, Health-related Quality of Life Instrument with 8 Items (HINT-8) score [9], General Anxiety Disorder 7-item scale (GAD-7) score [10, 11], stress score using a 4-point Likert scale, vHIT results, daily walking time, and daily sitting time.

### Definition of dizziness, examination guidelines for pure tone audiometry and vHIT, and items surveyed through a questionnaire

In the eighth KNHANES, the experience of dizziness was defined as experiencing dizziness or imbalance for a period of recent 12 months. And, chronic dizziness was defined as the recent experience of dizziness lasting for more than 3 months.

The otolaryngologic examinations were performed by four trained nurses affiliated with the Korean Society of Otorhinolaryngology-Head and Neck Surgery and were conducted for individuals aged 40 or over due to time and cost constraints.

Pure tone audiometry was conducted inside a 20-cm double-wall soundproof booth using an AD629 audiometer (Interacoustics, Assens, Denmark). Mean hearing threshold was calculated from the air-conduction hearing thresholds at 0.5 kHz, 1 kHz, 2 kHz, and 4 kHz.

The vHIT was performed to evaluate the lateral canal by administering more than 10 repetitive, unpredictable head impulse stimulations (100°–300°/second) using an EyeSeeCam system (Interacoustics, Middelfart, Denmark) while participants gazed at a point positioned 1.0 m away. Calibration was performed for each participant before conducting vHIT. And vHIT was conducted by trained nurses and reviewed by an otorhinolaryngology specialist. CUSs were defined as replicable rapid compensatory eye movements with an amplitude ≥100°/second and a latency ≤300 msec [12], and gain was determined by the slope of the linear regression equation between eye and head velocity for the 100 msec following the initiation of head movement. A VOR gain was calculated using regression methods, which calculate VOR gain with the average regression plot slope [13, 14]. VOR gain of less than 0.8 in either ear was considered abnormal [15]. We considered vHIT results abnormal when abnormal CUSs or VOR gain were observed.

The underlying diseases, HINT-8, GAD-7, stress score, daily walking time, and daily sitting time were collected through a survey.

### Classification of the participants based on their dizziness symptoms and vHIT results

We categorized the participants into groups based on their dizziness symptoms and vHIT results: an uncompensated group, displaying both abnormal vHIT result and chronic dizziness; a compensated group, displaying both abnormal vHIT result and a history of dizziness; an asymptomatic group, characterized by abnormal vHIT result but no history of dizziness; and a normal group, characterized by a normal vHIT result and no history of dizziness.

### Statistical analysis

Analysis of variance (ANOVA) and the chi-square test were used to compare individuals among the groups. Multivariate analysis of variance (MANCOVA) was conducted to calculate age- and gender-adjusted mean and standard error values and to detect significant associations. As post-hoc tests, the Bonferroni correction was applied for equal variance, the chi-square test, and MANCOVA; the Games-Howell test was applied for unequal variance. In addition, an ordinary logistic regression analysis was conducted to adjust the gender and age for the categorical values. A P-value less than 0.05 was defined as statistically significant. SPSS Statistics 25.0 (IBM Corp., Armonk, NY, USA) was used for all statistical analyses.

## Results

### Participants and their demographic factors, household income, and underlying diseases

**Fig 1:**
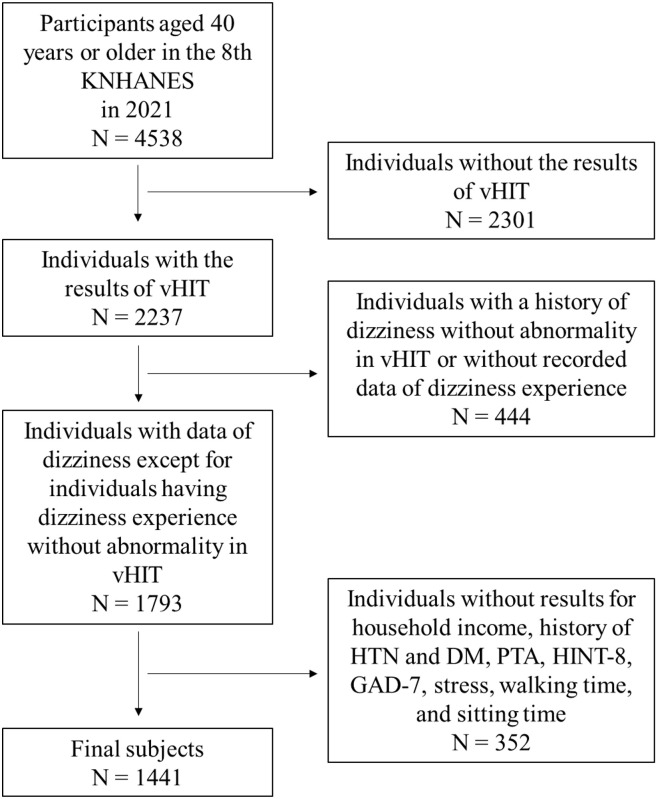
Flowchart showing the participant selection process. KNHANES, Korea National Health and Nutrition Examination Survey; N, number; vHIT, video head impulse test; HTN, hypertension; DM, diabetes mellitus; PTA, pure tone audiometry; HINT-8, Health-related Quality of Life Instrument with 8 Items; GAD-7, General Anxiety Disorder 7-item.

From the 4538 total participants aged 40 years or older in the eighth KNHANES in 2021, 2301 individuals with a history of cervical herniated disc, cataract surgery or intraocular lens insertion surgery, eyeliner tattoo or heavy eye makeup, as well as ptosis or those who refused vHIT, were excluded from the vHIT analysis. Subsequently, 444 individuals with dizziness and without vHIT abnormalities were excluded because their dizziness may have been caused by factors other than vestibulopathy. A further 352 individuals were excluded for lacking information on household income, history of dizziness, pure tone audiometry, HINT-8 score, GAD-7 score, stress score on a 4-point Likert scale, vHIT result, daily walking time, and daily sitting time. Finally, the total number of participants in our study was 1441 (Fig 1).

Since vHIT was conducted only for individuals aged > 40 years due to time and cost constraints, our study’s participants were middle-aged to elderly individuals, of which 681 were male and 760 were female. The mean age of the participants was 57.47 ± 10.88 years. Categorizing the participants into normal, asymptomatic, compensated, and uncompensated groups resulted in 1028, 302, 91, and 20 group members, respectively, with differing age (*P* < 0.001), gender (*P* = 0.001), and household income (*P <* 0.001) (Table 1). The normal group exhibited significantly younger age and higher household income compared to the uncompensated group (*P =* 0.008 for age, *P* = 0.010 for household income), compensated group (*P <* 0.001 for age, *P* = 0.005 for household income) or the asymptomatic group (*P <* 0.001 for age, *P* = 0.001 for household income). In addition, while the uncompensated group mostly consisted of women (85.00%, *P* = 0.004), the asymptomatic group predominantly contained males (53.97%, *P* = 0.009) compared to the participants as a whole. The prevalence of hypertension differed among the groups with statistical significance (*P* = 0.002) and was lowest in the normal group, whereas the difference in diabetes prevalence among the groups did not reach statistical significance (*P* = 0.267).

**Table 1:** Demographic factors, household income, underlying disease, mean hearing thresholds, and vHIT gain by group.

Factors	Groups				*P-value*
	Normal (N = 1028)	Asymptomatic (N = 302)	Compensated (N = 91)	Uncompensated (N = 20)	
**Demographics**					
Age (years)	55.84 ± 10.40	61.14 ± 11.09	62.33 ± 10.99	63.50 ± 9.69	***< 0*.*001***
Gender (male: female)	478: 550	163: 139	37: 54	3: 17	***0*.*001***
**Household income (quintile)**	3.44 ± 1.30	3.10 ± 1.39	2.96 ± 1.41	2.50 ± 1.15	***< 0*.*001***
**Mean hearing threshold (dB)**	18.55 ± 12.75	23.96 ± 15.96	24.48 ± 15.54	27.00 ± 13.94	***< 0*.*001***
**vHIT**					
Laterality of vestibulopathy (uni: bi)	-	185: 117	55: 36	14: 6	0.719
Catch up saccade (%)	0.00%	88.80%	79.07%	82.39%	***< 0*.*001***
Gain on the right side	1.06 ± 0.12	0.95 ± 0.18	0.98 ± 0.18	0.89 ± 0.19	***< 0*.*001***
Gain on the left side	1.05 ± 0.13	0.93 ± 0.18	0.95 ± 0.18	0.83 ± 0.22	***< 0*.*001***
The sum of the left and right gain	2.12 ± 0.22	1.88 ± 0.33	1.92 ± 0.32	1.73 ± 0.38	***< 0*.*001***

vHIT, video head impulse test; uni, unilateral; bi, bilateral;

* Mean hearing threshold was calculated from the air-conduction hearing thresholds at 0.5 kHz, 1 kHz, 2 kHz, and 4 kHz.

After controlling for age and gender, we found that the participants’ household income did not differ among the groups (*P* = 0.065, Table 2). Furthermore, no statistically significant distinction was found among the groups regarding hypertension and diabetes (*P* = 0.896 for hypertension, *P* = 0.583 for diabetes).

**Table 2:** Age-and gender-adjusted household income, mean hearing thresholds, and vHIT gain by group.

Factors	Groups				*P-value*
	Normal (N = 1028)	Asymptomatic (N = 302)	Compensated (N = 91)	Uncompensated (N = 20)	
**Household income (quintile)**	3.38 ± 0.04	3.25 ± 0.07	3.17 ± 0.13	2.81 ± 0.28	0.065
**Mean hearing threshold (dB)** *	19.86 ± 0.34	20.82 ± 0.62	20.94 ± 1.13	22.55 ± 2.40	0.234
**vHIT gain**					
Gain on the right side	1.06 ± 0.004	0.95 ± 0.01	0.98 ± 0.02	0.89 ± 0.03	***< 0*.*001***
Gain on the left side	1.05 ± 0.01	0.94 ± 0.01	0.95 ± 0.02	0.84 ± 0.03	***< 0*.*001***
The sum of the left and right gain	2.12 ± 0.01	1.88 ± 0.02	1.93 ± 0.03	1.73 ± 0.06	***< 0*.*001***

Data are presented as mean ± standard error. vHIT, video head impulse test;

* Mean hearing threshold was calculated from the air-conduction hearing thresholds at 0.5 kHz, 1 kHz, 2 kHz, and 4 kHz.

### vHIT test results and mean hearing threshold by group

The differences in the laterality of abnormal vHIT results were not statistically significant among the uncompensated, compensated, and asymptomatic groups (*P* = 0.719). The proportion of CUS was 0%, 88.80%, 79.07%, and 82.39% in the normal group, asymptomatic group, compensated group, and uncompensated group, respectively. Regarding the sum of right and left gain, vHIT gain differed among the groups (*P <* 0.001). The normal group had the highest sum of right and left gain at 2.12 ± 0.22 (*P* < 0.001). Additionally, the difference in the sum of right and left gain between the compensated group (gain = 1.92 ± 0.32) and uncompensated group (gain = 1.73 ± 0.38) was significant (*P* = 0.012). And, the sum of right and left gain of the asymptomatic group (gain = 1.88 ± 0.33) was not statistically significant different from that of compensated group (*P* = 1.000) or uncompensated group (*P* = 0.055) (Table 1). These tendencies were similar after controlling for age and gender (*P <* 0.001, Table 2).

The mean hearing threshold was lowest in the normal group (*P* < 0.001). However, after controlling for age and gender, we found no statistically significant difference in mean hearing threshold among the groups (*P* = 0.234, Table 2).

### HINT-8, GAD-7, and stress scores

**Fig 2:**
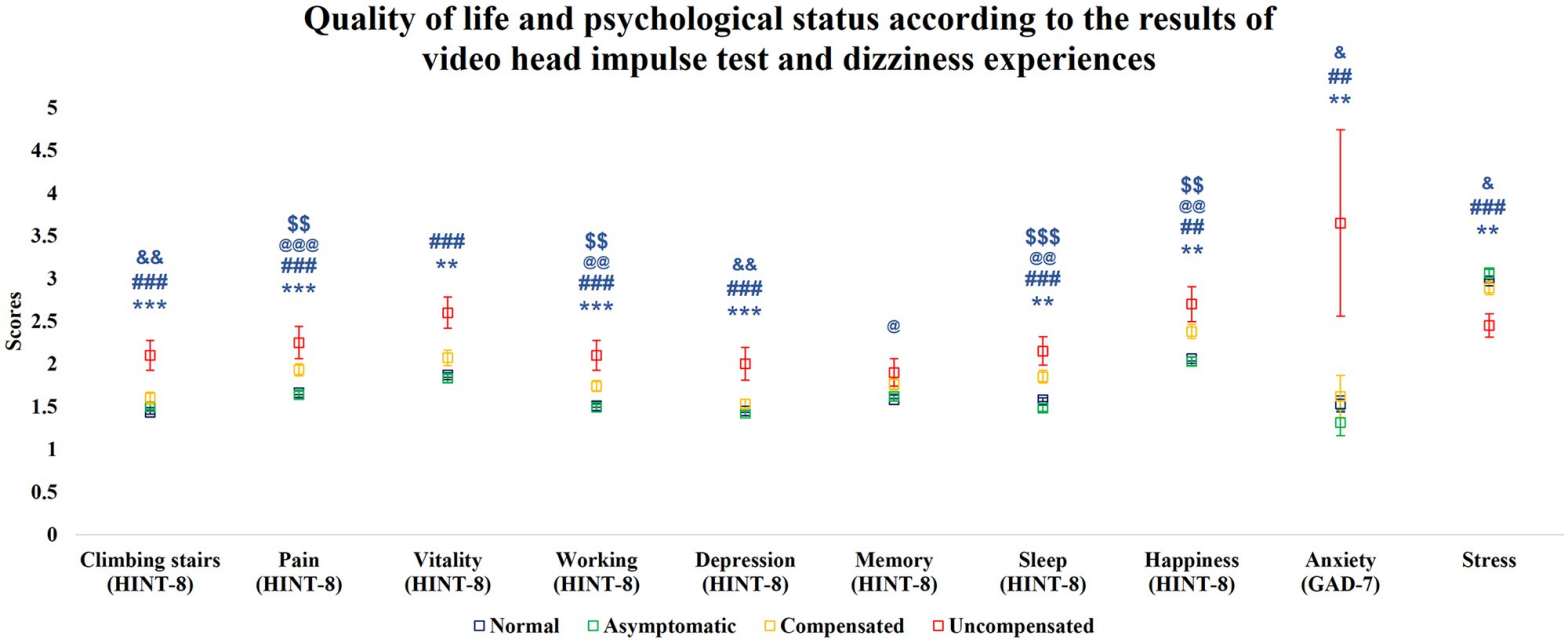
HINT-8, GAD-7, and stress (4-point Likert scale) scores by group. HINT-8, Health-related Quality of Life Instrument with 8 Items; GAD-7, General Anxiety Disorder 7-item. *, significant difference between the normal and uncompensated groups; *, *P <* 0.05; **, *P <* 0.01; ***, *P <* 0.001. @, significant difference between the normal and compensated groups; @, *P <* 0.05; @@, *P <* 0.01; @@@, *P <* 0.001. #, significant difference between the asymptomatic and uncompensated groups; #, *P <* 0.05; ##, *P <* 0.01; ###, *P* < 0.001. $, significant difference between the asymptomatic and compensated groups; $, *P <* 0.05; $$, *P <* 0.01; $$$, *P* < 0.001. &, significant difference between the uncompensated and compensated groups; &, *P <* 0.05; &&, *P <* 0.01; &&&, *P* < 0.001.

The HINT-8, GAD-7, and stress scores differed among the groups. The normal group and asymptomatic group did not show any difference in terms of subscales of HINT-8, GAD-7, and stress (Fig 2). The compensated group had higher HINT-8 scores for pain, work, sleep, and happiness than the normal group (*P* < 0.001 for pain, *P* = 0.006 for working, *P <* 0.001 for sleeping, *P* = 0.001 for happiness) and asymptomatic group (*P* = 0.001 for pain, *P* = 0.009 for working, *P <* 0.001 for sleeping, *P* = 0.001 for happiness). The compensated group also had higher HINT-8 score for memory compared to normal group (P = 0.023). The uncompensated group had higher HINT-8 scores (except for memory), GAD-7 scores, and stress scores than the normal group (*P* < 0.001 for climbing stairs, *P <* 0.001 for pain, P = 0.001 for vitality, *P* < 0.001 for working, *P* < 0.001 for depression, *P* = 0.001 for sleeping, *P* = 0.002 for happiness, *P* = 0.006 for anxiety, *P* = 0.003 for stress) and the asymptomatic group (*P* < 0.001 for climbing stairs, *P <* 0.001 for pain, *P* < 0.001 for vitality, *P* < 0.001 for working, *P* < 0.001 for depression, *P <* 0.001 for sleeping, *P* = 0.002 for happiness, *P* = 0.002 for anxiety, *P* < 0.001 for stress) (Fig 2). Furthermore, the uncompensated group had higher HINT-8 scores, GAD-7 scores, and stress scores than the compensated group in climbing stairs (*P* = 0.008), depression (*P* = 0.006), anxiety (*P* = 0.024), and stress (*P* = 0.046) (Fig 2).

**Fig 3:**
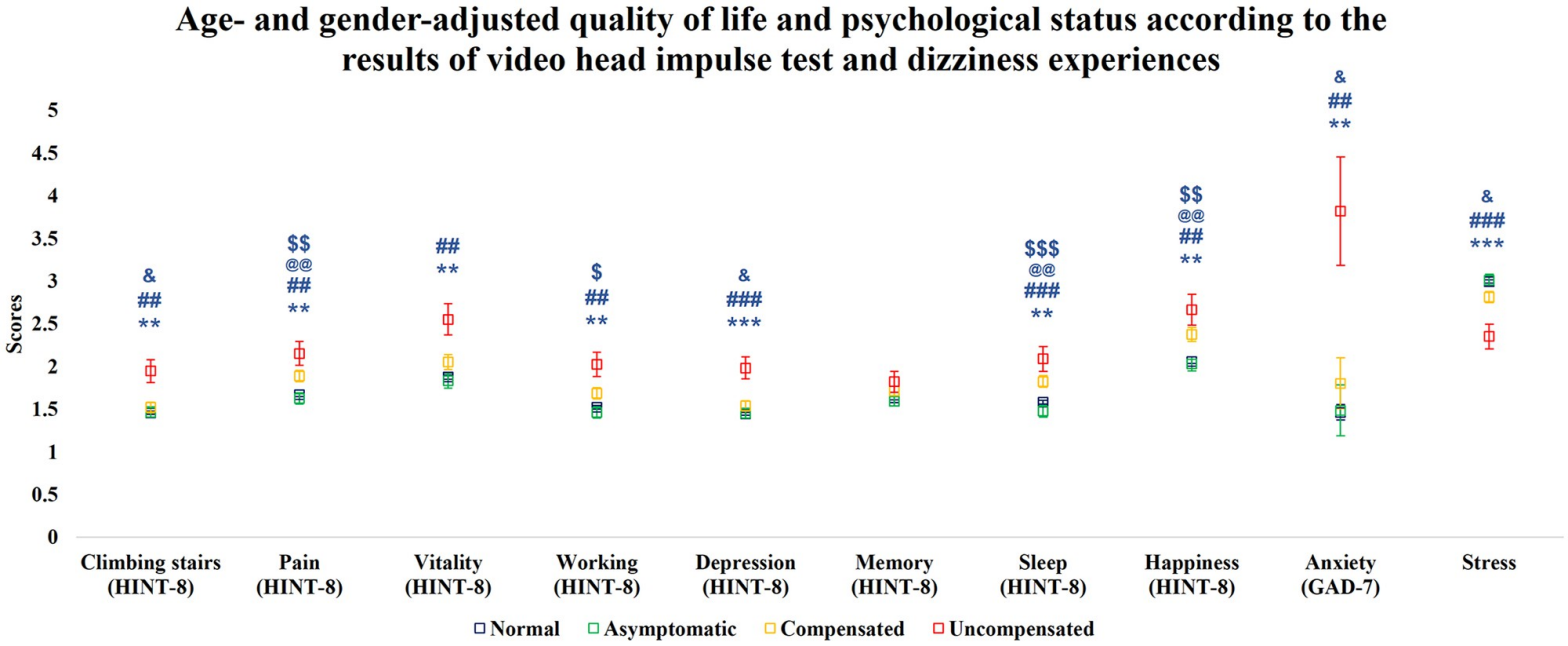
Age-and gender-adjusted HINT-8 and GAD-7 scores by group. HINT-8, Health-related Quality of Life Instrument with 8 Items; GAD-7, General Anxiety Disorder 7-item. *, significant difference between the normal and uncompensated groups; *, *P <* 0.05; **, *P <* 0.01; ***, *P <* 0.001. @, significant difference between the normal and compensated groups; @, *P <* 0.05; @@, *P <* 0.01; @@@, *P <* 0.001. #, significant difference between the asymptomatic and uncompensated groups; #, *P <* 0.05; ##, *P <* 0.01; ###, *P* < 0.001. $, significant difference between the asymptomatic and compensated groups; $, *P <* 0.05; $$, *P <* 0.01; $$$, *P* < 0.001. &, significant difference between the uncompensated and compensated groups; &, *P <* 0.05; &&, *P <* 0.01; &&&, *P* < 0.001.

Since household income, mean hearing threshold, and underlying diseases were not significantly different after adjustment for age and gender, only age and gender were included in the multivariable analysis. After controlling for age and gender, we found similar tendencies with unadjusted analyses among the HINT-8 (except for memory), GAD-7, and stress scores of each group (Fig 3).

### Physical activity by group

The differences among the groups in mean daily walking and sitting times did not achieve statistical significance in either the unadjusted analyses (*P* = 0.339 for walking time, *P* = 0.071 for sitting time) or the analyses adjusted for age and gender (*P* = 0.553 for walking time, *P* = 0.112 for sitting time) (Table 3).

**Table 3:** Daily walking and sitting times by group.

Factors	Groups				*P-value*
	Normal (N = 1028)	Asymptomatic (N = 302)	Compensated (N = 91)	Uncompensated (N = 20)	
**Unadjusted**					
Daily walking time (hours/day)	0.87 ± 0.80	0.91 ± 0.89	0.90 ± 0.72	1.18 ± 0.89	0.339
Daily sitting time (hours/day)	8.41 ± 3.23	8.51 ± 3.37	9.14 ± 3.19	9.70 ± 3.45	0.071
**Age-and gender-adjusted**					
Daily walking time (hours/day)	0.88 ± 0.03	0.88 ± 0.05	0.87 ± 0.09	1.14 ± 0.18	0.553
Daily sitting time (hours/day)	8.44 ± 0.10	8.43 ± 0.19	9.07 ± 0.34	9.71 ± 0.73	0.112

Unadjusted values are presented as mean ± standard deviation; Adjusted values are presented as mean ± standard error.

## Discussion

In this study, we demonstrated that uncompensated vestibulopathy was more common in older individuals and women. In addition, the uncompensated group had the poorest QOL and highest anxiety levels. The compensated group had a significant impairment in QOL compared to the normal or asymptomatic groups, despite the absence of current symptoms. In contrast, the asymptomatic group did not experience anxiety issues or reduced QOL. Although the groups did not differ significantly regarding physical activity as evaluated by their daily walking and sitting times, members of the uncompensated and compensated groups seemed to be concerned about their functional levels and to feel that QOL was impaired.

The participants with vestibular dysfunction tended to be older than the members of the normal group. In addition, the uncompensated group had a higher percentage of female members than the other groups. These results are consistent with previous studies that found that dizziness is more prevalent for women and that they are more susceptible to symptoms of dizziness [16–18]. Therefore, physicians should consider more intensive treatment, including vestibular rehabilitation therapy, for older women with vestibulopathy.

The uncompensated group with chronic dizziness experienced a generally impaired QOL, physical function, and emotional status. Our results were consistent with previous studies indicating that chronic dizziness can have significant effects on QOL, emotional status, and stress [2, 4, 19]. Compared to the uncompensated vestibulopathy group, the compensated group showed improved quality of life, physical function, and psychological health, including climbing stairs, depression, anxiety, and stress. Therefore, to improve QOL for these individuals, appropriate vestibular rehabilitation that includes sufficient support from visual and somatosensory cues is needed [7, 20, 21].

Compensated vestibulopathy was defined as vestibular hypofunction compensated by another balance-associated organ, such as the brain or eye [20, 22]. Given the high prevalence of vestibular vertigo, about 5% in one year and 7.5% for the lifetime of an individual, compensating for it and improving patients’ QOL is important [23, 24]. Our study showed a similar percentage affected by vestibulopathy (7.70%). Vestibular compensation can be facilitated by vestibular rehabilitation exercises [7, 20–22]. Although our study’s participants did not report dizziness at the time of the KNHANES survey and did not exhibit any deficits in physical function as evaluated by activities such as climbing stairs, daily walking time, and daily sitting time, they showed impairment in QOL, especially in terms of pain, working, sleeping, and happiness. After the participants’ vestibular hypofunction was compensated by visual and somatomotor sensory training, they tried to move en bloc with head and neck together to minimize the movement of the head alone, which can result in muscle fatigue and pain in the cervical region, as well as the regions along the spine [21]. In addition, reduced QOL might occur because the compensating process demands cognitive and emotional labor, which can impair other psychological activities [25]. Chronic pain, psychological costs, workplace performance, and sleep can mutually exacerbate each other, worsening each symptom [26–28]. Additionally, the possibility of experiencing severe dizziness could lead to a decline in QOL among members of the compensated group [29]. However, considering that their levels of depression, anxiety and stress were not significantly higher than those of normal or asymptomatic group members, their impairment in QOL might be less closely associated with earlier traumatic experiences due to dizziness. To improve their QOL, sustained vestibular rehabilitation testing is necessary even after their symptoms improve [20, 21], together with treatment for their physical symptoms, such as pain and sleep issues.

Contrary to other domains of QOL, neither the compensated nor the uncompensated participants complained of memory impairment in the multivariable analysis. Some previous studies have demonstrated that spatial memory is impaired in individuals with vestibulopathy [30–32]. However, the association between vestibulopathy and other subcategories of memory remains a matter of debate [32–34]. In our findings, although dizziness imposed a cognitive and emotional burden, it did not seem to be significantly associated with overall memory. However, since our results were derived from a small number of uncompensated patients without exact information on the cause, duration, and characteristics of their dizziness, further studies with precise information on uncompensated vestibulopathy and a larger sample size are necessary to clarify the association between uncompensated vestibulopathy and memory.

Among our study’s participants, asymptomatic vestibular dysfunction was usually identified in elderly individuals or individuals with vestibular schwannoma [35]. Our results showed that the asymptomatic group did not have any complaints regarding impaired QOL despite similar results of vHIT gain to those of patients with compensated vestibulopathy. This might be because of the slow, progressive course of asymptomatic vestibulopathy. Due to its slow progression, asymptomatic vestibulopathy may be compensated by other sensory organs without discomfort [36]. Therefore, treatment may not be necessary for asymptomatic patients with incidental vestibulopathy.

In our study, the size of the uncompensated group was small. We could not include more participants because we extracted data from an existing database. For a more accurate evaluation of QOL features, physical status, and psychological status among uncompensated individuals, further investigation with a larger group would be helpful.

One limitation of our study was the absence of results from other vestibular workups. Since BPPV, persistent postural perceptual dizziness, or other functional dizziness can accompany vestibular dysfunction, the inclusion of results from additional vestibular workups would help identify the exact diagnosis for dizziness. Furthermore, we did not have an exact diagnosis at the time of the dizziness for the subjects. Vestibulopathy can be caused by various diseases, such as AUVP, Meniere’s disease, and vestibular schwannoma [1]. Meniere’s disease tends to involve recurrent vestibulopathy, as well as a hearing deficit and an association with migraine [37]. The different clinical features of the causes of vestibulopathy could act as covariates. Further studies with more comprehensive vestibular workups and an exact diagnosis for each participant’s dizziness are necessary to investigate the precise QOL, psychological, and physical status in individuals with compensated or uncompensated vestibulopathy.

Another limitation of this study is that the characteristics of dizziness and treatment for dizziness were not fully described. Because dizziness was defined as an experience of dizziness or imbalance within the past year, and the duration of dizziness was classified only as less than 3 months or 3 months or more in the 8^th^ KNHANES, we could not obtain exact information on the characteristics of dizziness and its exact duration. Additionally, treatment and past medical history related to dizziness are not described in the KNHANES. Treatment for vestibulopathy, including vestibular rehabilitation [21], can improve vestibular symptoms and adjustment for chronic imbalance. Further studies with detailed information on the characteristics and medical history of dizziness may be helpful in assessing the QOL for individuals with vestibulopathy.

## Conclusion

Uncompensated vestibulopathy was found to be more common in older individuals and women. Although the degree of physical function was similar among participants, vestibulopathy with the experience of dizziness had a long-lasting impact on QOL and happiness even after it was compensated, and chronic dizziness had a more significant effect on QOL and psychological health. In contrast, the asymptomatic group showed no decrease in QOL and mental health. Interventions appropriate to each category of patients are necessary to improve their QOL and psychological health.
